# Exophytic hepatocellular carcinoma, simulating a mesenchymal tumor, in a non-cirrhotic liver

**DOI:** 10.1590/0100-3984.2015.0118

**Published:** 2017

**Authors:** Glaucio Rodrigo Silva de Siqueira, Marcos Duarte Guimarães, Luiz Felipe Sias Franco, Rafaela Batista e Silva Coutinho, Edson Marchiori

**Affiliations:** 1 Hospital Heliópolis, São Paulo, SP, Brazil.; 2 A.C.Camargo Cancer Center, São Paulo, SP, Brazil.; 3 Universidade Federal do Rio de Janeiro (UFRJ), Rio de Janeiro, RJ, Brazil.

Dear Editor,

A 26-year-old female presented with a five-month history of epigastric pain, nausea, and vomiting. She had recently lost weight (7 kg in the last month). Upon clinical examination, a bulky mass was palpated in the epigastric region.

**Figure 1:**
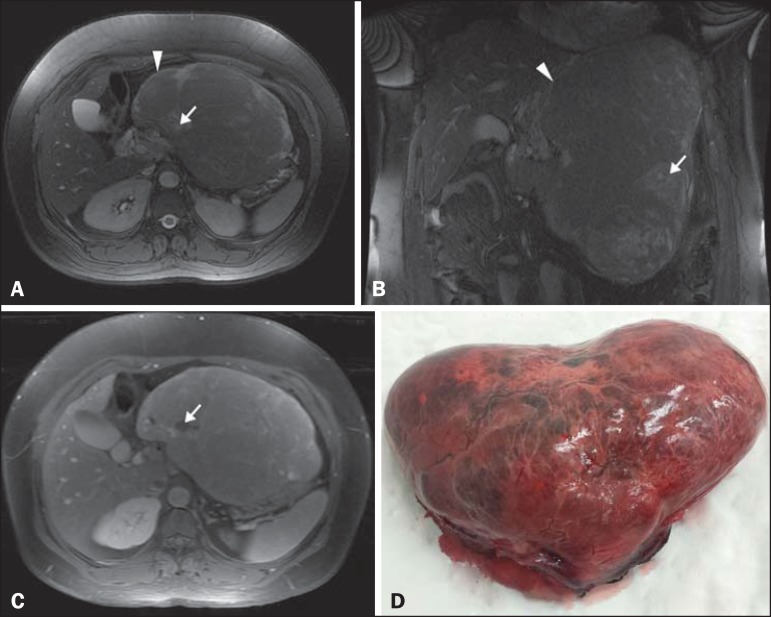
**A,B:** Axial and coronal fast imaging employing steady-state acquisition MRI with fat suppression. Solid, encapsulated, heterogeneous expansive mass in the epigastrium (arrowhead), with lobulated contours and areas of hyperintensity (arrow) consistent with necrosis. **C:** T1-weighted MRI acquisition with fat suppression after intravenous administration of paramagnetic contrast. Diffuse, heterogeneous paramagnetic contrast uptake by the neoplasm. Note the areas without uptake, which is consistent with necrosis (arrow). **D:** Macroscopic examination. Specimen received in formalin, designated as the product of a left hepatectomy, consisting of a liver fragment weighing 2354 g and measuring 23 × 17 × 11 cm, with an irregular shape, a smooth, brownish external surface, and a bloody area that measured 10 × 6 cm.

Magnetic resonance imaging (MRI) (Figures 1A, 1B and 1C) revealed a solid, encapsulated, heterogeneous expansive mass in the epigastrium. The mass showed lobulated contours, measured approximately 25 × 20 × 12 cm, and had a volume of 3120 cm^3^. Within the mass, which was compressing the body and tail of the pancreas, as well as the splenic vein, gastric fundus, and left lobe of the liver, there were foci of hyperintensity on T2-weighted images and hypointensity on T1-weighted images. The lesion presented discrete heterogeneous paramagnetic contrast enhancement. The results of laboratory tests, including alphafetoprotein levels, were within the limits of normality.

The patient underwent left lobe hepatectomy and resection of the neoplasm (Figure 1D). Pathological examination revealed a multifocal, Edmondson-Steiner grade II hepatocellular carcinoma, with macrotrabecular components, that was pseudoacinar and contained clear cells (moderately differentiated hepatocellular carcinoma).

Hepatocellular carcinoma is the most common primary tumor of the liver^(1)^, although several other histological types have been reported^(2-5)^. Although hepatocellular carcinoma typically occurs in patients with liver cirrhosis, approximately 20% of cases occur in patients without it^(6)^. Its incidence peaks in the second and seventh decades of life, and it affects twice as many men as women^(6)^. Although hepatocellular carcinoma presents a variable aspect on MRI, it is typically hyperintense or isointense on T2-weighted images, whereas it is typically hypointense on T1-weighted images^(7,8)^. After administration of paramagnetic contrast, hepatocellular carcinoma presents intense enhancement in the arterial phase and hypointense signals in the portal and equilibrium phases, characterizing the contrast medium washout pattern^(9)^. Tumors larger than 1.5 cm typically present a fibrous capsule that appears as a hypointense band in the late phases^(8,9)^. Occasionally, hepatocellular carcinoma manifests as a large solitary mass^(1,8)^.

Exophytic/pedunculated hepatocellular carcinoma is extremely rare^(10)^. One study showed that this type of tumor accounts for 0.24-3.0% of all cases of hepatocellular carcinoma in Japan^(11)^. It has an atypical presentation, manifesting as an extra-hepatic mass in imaging studies, simulating another type of primary tumor^(12)^. In another study, there is a report of seven patients with extrahepatic masses seen on computed tomography, all simulating tumors of primary extrahepatic origin, in which the diagnosis of exophytic hepatocarcinoma was established only after percutaneous biopsy, surgical resection, or necropsy^(13)^.

Here, we have presented the case of a patient who was young, had no history of liver disease or known risk factors for liver cirrhosis, had normal alpha-fetoprotein serum levels, and presented with a large epigastric mass that showed a hypovascular contrast pattern and was in contact with the liver. The main diagnoses considered were mesenteric sarcoma and an epithelioid gastrointestinal stromal tumor. In accordance with the findings of other studies, the diagnosis could not be made solely on the basis of the clinical data and MRI images obtained.

Exophytic hepatocellular carcinoma is difficult to diagnose. Therefore, when a bulky mass is discovered and is in contact with the surface of liver, this diagnostic possibility should be considered, even in patients who do not present risk factors for the condition^(14)^.
